# Barriers and Determinants of Referral Adherence in AI-Enabled Diabetic Retinopathy Screening for Older Adults in Northern India During the COVID-19 Pandemic: Mixed Methods Pilot Study

**DOI:** 10.2196/67047

**Published:** 2025-03-31

**Authors:** Anshul Chauhan, Anju Goyal, Ritika Masih, Gagandeep Kaur, Lakshay Kumar, ­ Neha, Harsh Rastogi, Sonam Kumar, Bidhi Lord Singh, Preeti Syal, Vishali Gupta, Luke Vale, Mona Duggal

**Affiliations:** 1 Advanced Eye Centre, Post Graduate Institute of Medical Education and Research, Number 139, Kairon Block, Chandigarh, 160012, India, 91 9560374388; 2 Department of Statistics, Panjab University, Chandigarh, India; 3 Newcastle University, Newcastle, United Kingdom; 4 National Institute for Research in Digital Health and Data Sciences, Indian Council of Medical Research, New Delhi, India

**Keywords:** diabetic retinopathy, diabetes, gerontology, geriatric, old, aging, aged, artificial intelligence, retinopathy, retinal, referral, screening, optometry, ophthalmology, adherence, barriers

## Abstract

**Background:**

Diabetic retinopathy (DR) is a leading cause of blindness globally. DR has increasingly affected both individuals and health care systems as the population ages.

**Objective:**

This study aims to explore factors and identify barriers associated with nonadherence to referral recommendations among older adult participants after DR screening (DRS) during the COVID-19 pandemic.

**Method:**

This paper presents findings from a pilot study on artificial intelligence–enabled DRS conducted in two districts in Punjab, India (Moga and Mohali) during the COVID-19 pandemic. The screenings were conducted from March to June 2022 at community health center Badhani Kalan in Moga and from March to June 2021 in community settings (homes) in Block Boothgarh, Mohali. Participants were referred to the district hospital for an ophthalmological review based on artificial intelligence–enabled screening. After 1 month, the participants were contacted by telephone to assess adherence to the referral recommendations. Participants who did not adhere to the referral were then interviewed alongside health care providers to understand the barriers explaining their nonadherence.

**Results:**

We aimed to recruit 346 and 600 older adult participants from 2 sites but enrolled 390. Key challenges included health facility closures due to COVID-19, low motivation among health personnel for recruitment, incomplete nonparticipation data, and high participant workloads. Approximately 45% of the participants were male and 55% female. Most participants (62.6%) were between 60 and 69 years old, while 37.4% were 70 or older, with a mean age of 67.2 (SD 6.2) years. In total, 159 participants (40.8%) were referred, while 231 participants (59.2%) were not. Only 23 (14.5%) of those referred followed through and visited a health facility for ophthalmological review, while 136 (85.5%) did not pursue further evaluation. Our analysis revealed no significant differences in the characteristics between adherent and nonadherent participants, suggesting that demographic and health factors alone do not predict adherence behavior in patients with DR. Interviews identified limited knowledge about DR, logistical challenges, financial constraints, and attitudinal barriers as the primary challenges.

**Conclusions:**

This study, conducted during the COVID-19 pandemic, showed suboptimal adherence to referral recommendations among older adult patients due to knowledge gaps, logistical challenges, and health system issues. Quantifying and understanding adherence factors are crucial for targeted interventions addressing barriers to referral recommendations after DRS. Integrating teleophthalmology into and strengthening infrastructure for artificial intelligence–enabled diabetic retinopathy screening to enhance access and outcomes.

## Introduction

Diabetic retinopathy (DR) is a prevalent and severe microvascular complication of diabetes mellitus (DM) [1]. In India, 10.9% (7.2%‐16.3%) of individuals aged 65 years and above with diabetes have DR, with 2.3% (1.2%‐4.4%) suffering from vision-threatening diabetic retinopathy (VTDR) [1], characterized by severe retinopathy or macular edema [2]. DR is typically asymptomatic in its early stages, and it can lead to visual impairment or blindness if left untreated [3]. The rate of blindness due to VTDR is expected to rise proportionately to the exponential increase in DM prevalence [4,5]. Visual impairment due to DR increases by 9.6% in individuals over 50 [6], emphasizing advancing age as a significant risk factor [7]. Individuals with VTDR face greater difficulties in vision-related tasks, such as reading, watching television, and driving [8]. Additionally, people with DR have a higher risk of falls, resulting in reduced mobility, social isolation, and increased physical dependence [9].

India is set to become home to the world’s second-largest older adult population, with the number of people over 60 years of age projected to rise from 100 million in 2013 to 198 million by 2030 [10]. The National Programme for Health Care of the Elderly in India advocates for accessible, affordable, high-quality, long-term, comprehensive care services tailored to the needs of an aging population [10]. Significant scientific evidence shows that early screening and timely treatment referral can prevent most visual loss caused by DR [11]. Conventionally, DR screening (DRS) includes fundus (retina) examination by ophthalmologists or color fundus photography using conventional cameras (mydriatic or nonmydriatic) conducted by trained eye technicians or optometrists [12]. However, the sharp rise in diabetes cases, coupled with a shortage of trained retinal specialists and ophthalmologists [13], makes DRS services accessibility challenging, particularly as 80% of India’s older adult population resides in rural areas [10]. The COVID-19 pandemic posed additional significant challenges to health care systems worldwide, inevitably leading to the curtailment of health services accessibility, including DRS [14,15].

Automated computer-based analysis of fundus images could ease the strain on health care systems by streamlining DRS and offering a more efficient management solution [13]. Artificial intelligence (AI) systems have reduced costs, enhanced diagnostic precision, and expanded patient accessibility to DRS services [16] and immediate AI-supported feedback on referral status was linked to higher referral adherence [17].

Previous studies have demonstrated telemedicine-based DRS programs’ effectiveness, scalability, and sustainability in improving accessibility and reducing disparities [18]. The COVID-19 pandemic further emphasized the need for AI-enabled DRS and teleconsultations to standardize workflows and reduce human interactions to mitigate transmission risks [19,20].

These studies are particularly relevant for designing and implementing effective AI-enabled DRS in India, where challenges of health care accessibility and resource constraints exist [20]. Suboptimal adherence to follow-up recommendations impacts treatment outcomes and can undermine even the most effective treatment options [21]. A multicenter analysis showed a decline in laser procedures and vitrectomies for retinal detachment repair due to reduced clinic visits during the pandemic [22]. Patients faced barriers to follow-up eye exams, including transportation issues, high costs, and long wait times [23]. Programs such as a point-of-care DR examination program highlight the potential of telemedicine in reducing barriers to DRS, though adherence to follow-up recommendations after screening remains a major challenge [24].

However, some factors (individual, health) influencing adherence and nonadherence to referral services remain unknown [25]. During the COVID-19 pandemic, patients were prompted to limit hospital visits, minimize time spent there, and reduce direct contact with health care providers (HCPs). Limited research exists in India on older adults’ adherence to DRS referral recommendations and the barriers influencing their decisions.

**Figure 1.**
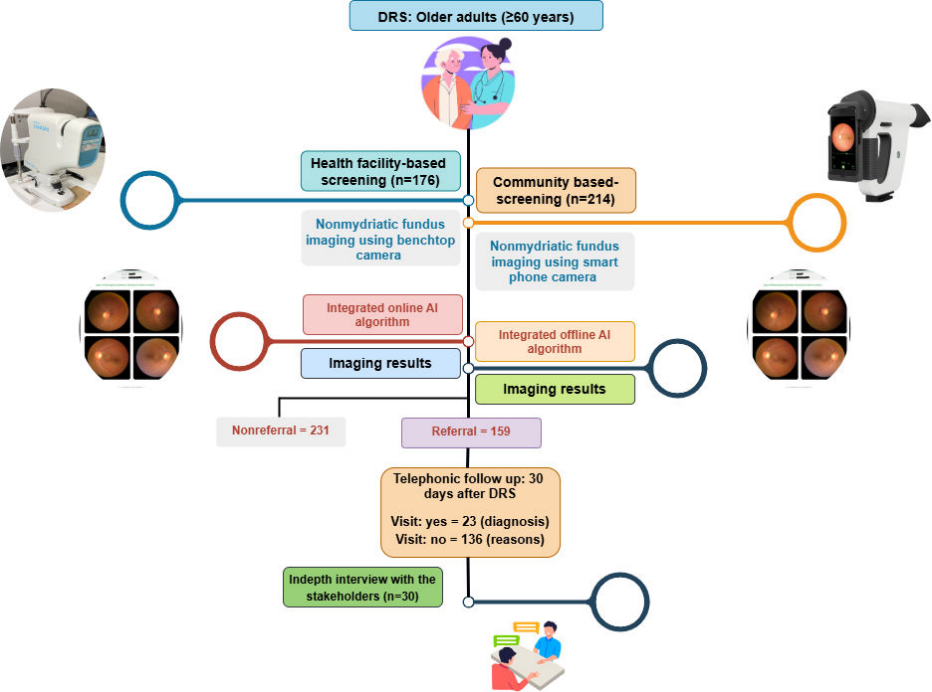
Overall study design. AI: artificial intelligence; DRS: diabetic retinopathy screening.

This study aims to (1) evaluate the referral adherence rates among older adult participants after AI-enabled DRS and identify the key factors affecting adherence during the COVID-19 pandemic and (2) identify and analyze the barriers to adherence to referral recommendations during the COVID-19 pandemic. The overall study design is described in Figure 1.

## Methods

### Study Setting and Participants

#### Quantitative

This paper presents findings from 2 districts in Punjab, India: Moga and Mohali. Screenings in Moga occurred at the community health center (CHC) Badhani Kalan from March to June 2022, while in Mohali, they were conducted in community settings (home) in Block Boothgarh from March to June 2021. The study included older adults [10] aged 60 years and above with a history of DM at both sites. Participants without a history of DM, those unwilling to provide informed consent, and those with a history of intraocular surgery, conjunctivitis, red eye, injury, or any eye inflammation were excluded.

A nurse at the medicine clinic at the CHC, Badhani Kalan, in district Moga, identified the elderlyolder adult participants with DM for DRS. In district Mohali, the list of diabeticparticipants with diabetes was obtained from thePa primary Hhealth Ccentere (PHC), in Boothgarh, and approached with the help of the village’s Accredited Social and Health Activists (ASHA) worker. The participants agreeing to undergo DRS received an appointment card, and a reminder telephone call was placed a day before the screening to confirm their attendance at home.

The repeated lockdowns during the COVID-19 pandemic disrupted health care services, significantly increasing the burden on health systems. Consequently, recruitment efforts were hindered, making it difficult to reach the target sample sizes of 348 at CHC Badhani Kalan and 600 at Block Boothgarh. Since the populations shared similar health systems and implementation challenges, the data from both sites were pooled and presented. Consequently, data pooling enhanced sample size and statistical power and effectively addressed research questions [26].

#### Qualitative

The in-depth interviews were conducted with 2 distinct categories of participants: older adults and HCPs. Older adult participants who did not adhere to the recommended referral instructions were purposely sampled and approached for in-depth interviews to understand their barriers to referral adherence after DRS. The nurse at the ophthalmology clinic invited older adult participants to the CHC for in-depth interviews, while ASHA workers contacted older adult participants in the community to conduct interviews at homes.

The second group of participants, HCPs, were purposely selected according to their roles in diabetes and DR diagnosis, referral, and treatment (Table S1 in Multimedia Appendix 1). The inclusion criteria were having at least 3 years of experience working in public health care settings and a willingness to describe patient experiences and barriers to referral adherence (Table S2 in Multimedia Appendix 1). The HCPs included ASHAs from the community settings [27], Community Health Officers (CHOs) from health and wellness centers [28], optometrists from a CHC [29] , ophthalmologists from the district hospital [30], and retina specialists from the retina unit of a tertiary eye care hospital.

Semistructured interviews were conducted to explore participants perspectives on the barriers to adhering to referral recommendations following AI-enabled DRS. The in-depth interview guides were developed in English, informed by existing literature on barriers to DR screening and referral [31-34], and later translated into Hindi and Punjabi (Table S3 in Multimedia Appendix 1). These guides, featuring open-ended questions and probes, explored participants’ perspectives on referral adherence after DRS. Pilot interviews with 2 people with DM, an ASHA, and a CHO led to revisions for clarity, replacing ambiguous terms with simpler language for better participant understanding.

### Ethical Considerations

Written informed consent was obtained from all participants, with both studies approved by the Institutional Ethics Committee of the Post Graduate Institute of Medical Education and Research, Chandigarh, India (PGI/IEC/2020/000741 and PGI/IEC/2020/001342). To ensure patient privacy and confidentiality, all images and associated metadata were de-identified prior to analysis. The studies adhered to the Declaration of Helsinki guidelines, and the study protocol is registered with the Clinical Trials Registry, India (CTRI 2022/10/046283 and 2022/10/046185).

### Hardware and Software for DRS

#### Hardware

The data were collected from 2 locations across districts in Punjab, each employing a different DRS methodology. Health facility–based screening at CHCs requires desktop nonmydriatic fundus cameras, while community screenings (home) rely on portable cameras for enhanced accessibility.

#### Fundus Benchtop Camera

The screening at the CHC Badhani Kalan utilized the Forus 3 Nethra classic (benchtop) camera, supported by a laptop running MS Windows 10 or higher, featuring a 64-bit operating system and an i3 10th-generation processor [35,36].

#### Smartphone-Based Fundus Camera

The lightweight, portable Fundus on Phone Non Mydriatic 10 (Remidio) handheld device, with a power backup, was used for screening in community settings in Block Boothgarh [37].

#### Software

Both sites used validated AI screening algorithms for DRS. At the CHC, an online DRS algorithm (Revelo) was integrated into the benchtop fundus camera [38]. A camera-integrated offline AI algorithm (Medios) was used for community-based screening [37,39].

### Data Collection

#### Fundus Photography Protocol

##### Training Protocol

Before patient recruitment, 2 optometrists underwent 15 days of 1-on-1 training at the Advanced Eye Centre of Post Graduate Institute of Medical Education and Research, Chandigarh, and a primary health care center in Block Boothgarh of district Mohali. The training covered recruitment and consenting procedures, history recording, retinal image acquisition, troubleshooting poor-quality images, automated grading with the AI system, and delivering grading reports to patients. Training and supervision continued until staff were comfortable independently collecting data and performing imaging.

##### Image Acquisition and Grading

Adequate ventilation was maintained for patient consultations and fundus photography. Optometrists wore N95 masks and followed strict hand hygiene. Patients without masks were required to wear one during interactions and in waiting areas. Optometrists obtained nonmydriatic macula and disc-centered image color fundus photographs at 45° field of view using low-cost cameras (Figure 1) for participants at both sites. The screening at the CHC was carried out in an ophthalmology clinic. On the day of screening, the optometrists were assisted by ASHA workers at the homes of the eligible participants. The AI algorithms provided results at both sites. Two human graders, a certified optometrist and an ophthalmologist, graded all the fundus images. A senior retina specialist resolved any disagreements between the graders. All the classifications of DR were based on the International Classification for Diabetic Retinopathy [40].

##### Referral and Telephonic Follow-Up

Participants received an AI-generated report detailing their DR status, severity, and referral recommendations. Patients diagnosed with moderate non-proliferative DR and above were referred to as referable DR [41] and, with ungradable images, were advised to visit the district hospital for an ophthalmological review. One month after the screening, participants were followed up with telephone calls to confirm adherence and obtain diagnosis details. Each participant was contacted thrice, on 3 consecutive days, before being classified as nonresponsive. A structured questionnaire was administered to participants via telephone. The questionnaire assessed factors influencing appointment attendance and treatment adherence, with responses recorded as yes, no, or do not know for each question [42,43]. The questionnaire assessed sociodemographic characteristics and perceived barriers to referral (knowledge, attitude, logistics) and included an open-ended question for unlisted reasons behind incomplete referrals.

### In-Depth Interviews

Two research fellows (AC and HR) with master’s degrees and over 5 years of qualitative research experience conducted the in-depth interviews in the participant’s preferred languages (Hindi, Punjabi, and English). Participants with DM were interviewed in quiet hospital locations at the CHC or at home in a properly ventilated space while HCPs were interviewed face-to-face or via Zoom, based on availability. Study procedures were explained, and informed consent was obtained, either written for in-person interviews or verbal for Zoom sessions. Each interview was audio recorded and lasted for 35‐40 minutes. The final sample size for the qualitative study was determined based on the principle of data saturation [44,45].

### Data Analysis

Using a mixed methods approach, the results from both the quantitative and qualitative components were compared, synthesized, and discussed.

#### Quantitative

All participant data were entered into Research Electronic Data Capture (REDCap) [46], exported in Excel format, cleaned, and subsequently analyzed using Stata/IC (version 14.2; StataCorp LLC). The analysis involved reporting the descriptive statistics. Histograms were used to assess the normality of continuous variables. Summary statistics include frequencies (percentages) for categorical variables and means (standard deviations) for normally distributed continuous variables; otherwise, medians and interquartile ranges were reported. A chi-square (*Χ*^*2*^) test was conducted to compare the distribution of categorical variables (eg, gender, education, marital status) between the adherent and nonadherent participant groups.

Univariate logistic regression was used to estimate odds ratios (ORs) and 95% CIs for the association between each independent variable (eg, gender, age, education, occupation, marital status, income, health insurance, duration of diabetes, hypertension status, and previous eye facility visits) and the dependent variable (referral adherence status: adherent versus nonadherent). The multivariate logistic regression model included independent variables with a *P* value <.05 in univariate analysis to adjust for potential confounding factors and identify independent predictors of referral nonadherence, using a significance level of *P*<.05 for the final model.

#### Qualitative

The qualitative researchers proficient in Hindi and Punjabi transcribed the recordings verbatim, translated them into English, and removed all personal identifiers before analysis. We analyzed the interview transcripts using thematic analysis, following Braun and Clarke’s 6-phase approach [47]. Although the phases progressed logically, the process was not linear but recursive and iterative, involving movement back and forth between phases as needed [47]. We systematically organized the data, coded it, and grouped similar codes into themes to identify barriers to referral adherence. The research team members (AC: research scholar; RM: research associate; HR: research scholar) performed independent open transcript coding. MD, an experienced public health specialist, reviewed and confirmed the initial coding framework. Coding and categorization under themes were done using Atlas.Ti 23 software.

We then began extracting relevant portions of text from each interview related to the categories, sorting and selecting quotes and placing them under the appropriate themes. Quotes were edited for readability to retain their original meaning; ellipses (...) show removed text. The participants are denoted in italics with a unique ID number and their group designator: P (people with DM), Opt (Ophthalmologists), ASHA (ASHA workers), CHO (Community Health Officer), Optom (Optometrists), and RS (Retina Specialist). Given that both study sites are in Punjab and share similar health system building blocks [48], the in-depth interviews were analyzed and presented collectively.

## Results

### Quantitative

#### Participant Characteristics

A total of 390 (41%) of 948 older adult participants were enrolled across both sites, with 176 (45%) males and 214 (55%) females, primarily due to COVID-19 restrictions and participant refusals. Participants aged 60‐69 years comprised 62.6244% of the sample, while those aged ≥70 years constituted 37.4%, with a mean age of 67.2 (SD 6.2) years. Around 48.5% had no formal education, 45.9% had education up to the 10th standard, and 5.6% had education at the 12th standard or higher. Occupationally, 69.7% were unemployed or engaged in home duties, 13.8% were retired, and 16.5% were in other categories. Marital status indicated that 74% were married. Additionally, 69% had a monthly household income below 30,000 Indian rupees (INR; approximately US $350), 52.3% had a history of hypertension, and the mean DM duration was 8.03 (SD 6.9) years. Site-wise demographic details are available in Table S4 in Multimedia Appendix 1.

#### Referral Recommendations and Adherence Rates

Out of 390 screened participants, 231 (59.2%) were not referred, while 159 (40.8%) were referred for further review and management. Among those referred, only 23 (14.5%) followed the advice and visited a health facility (details in Table S5 in Multimedia Appendix 1 ), while 136 (85.5%) did not adhere to the referral recommendations or seek further medical attention.

#### Differences Between Referral Categories (Adherent and Nonadherent)

Table 1 shows the demographic and medical characteristic differences between adherent and nonadherent participants in the referral category. The comparison of 159 referral participants showed no statistically significant differences in demographics and health characteristics between adherent (n=23) and nonadherent (n=136) groups (all *P*>.05). This includes gender distribution (*P*=.23), age groups (*P*=.48), educational levels (*P*=.27), occupational status (*P*=.33), marital status (*P*=.97), household income (*P*=.71), health insurance coverage (*P*=.93), duration of DM (*P*=.51), presence of hypertension (*P*=.16), and previous eye facility visits (*P*=.45).

**Table 1.** Difference in demographics and health characteristics between adherent and nonadherent participants in the referral category.

Variable	Referral category (n=159)		
Adherent (n=23)	Nonadherent (n=136)	*P* value
**Gender, n (%)**			.23
Male	12 (52.7)	53 (39)	
Female	11 (47.8)	83 (61)	
**Age group (years), n (%)**			.48
60‐69	14 (60.8)	72 (53)	
≥70	9 (39.1)	64 (47)	
**Education, n (%)**			.27
No formal education	8 (34.8)	72 (53)	
Up to 10th standard	14 (60.8)	59 (43.4)	
12th standard and above	1 (4.4)	5 (3.6)	
**Occupation, n (%)**			.33
Retired from service	4 (17.4)	15 (11)
Unemployed/home duties	14 (60.9)	103 (75.8)
Other	5 (21.7)	18 (13.2)	.97
**Marital status, n (%)**			
Married	16 (69.5)	94 (69)	
Unmarried, divorced, separated, widow, widower	7 (30.4)	42 (31)	
**Monthly household income (INR)^a^, n (%)**			.71
<30,000	15 (65.2)	94 (69)
>30,000	8 (34.8)	42 (31)
**Health insurance, n (%)**			.93
Yes	8 (34.8)	46 (33.8)
No	15 (65.2)	90 (66.2)
**Duration of DM^b^ (years), n (%)**			.51
0‐10	17 (73.9)	91 (67)
≥10	6 (26.1)	45 (33)
**Hypertension (years), n (%)**			.16
Yes	15 (65.2)	67 (49.2)
No	8 (34.8)	69 (50.8)
**Previous visit to the eye facility, n (%)**			.45
Once	19 (82.6)	120 (88.2)
Never	4 (17.4)	16 (11.8)

a INR: Indian rupee. 30,000 INR=US $350.

b DM: diabetes mellitus.

### Analysis of Determinants for Nonadherence to Referral Recommendations

Table 2 compares determinant factors between 23 adherent and 136 nonadherent participants using univariate and multivariate logistic regression analyses. None of the variables showed statistically significant differences (all *P*>.05), indicating no evidence of associations with adherence status across the examined factors. Gender (female) showed nonsignificantly higher odds of nonadherence, for univariate (OR 1.7, 95% CI 0.7-4.2*; P*=.23) and multivariate (OR 1.07, 95% CI 0.31-3.72*; P*=.91). Age ≥70 years had univariate and multivariate ORs of 1.4, 95% CI 0.56-3.4 *(P*=.48) and 1.4, 95% CI 0.5-4.09 *(P*=.47), respectively, indicating no significant association with adherence. Similarly, no significant differences were found in occupation categories, marital status, household income, health insurance status, duration of DM, hypertension, or previous eye facility visits (all *P*>.05).

**Table 2.** Univariate and multivariate analysis of the determinants for nonadherence to referral recommendations. Reference: Base category used for comparison.

Variable	Adherent (n=23)		Nonadherent (n=136)	
	Univariate OR^a^ (95% CI)	*P* value	Multivariate OR (95% CI)	*P* value
**Gender, n (%)**				
Male	Reference		Reference	
Female	1.7 (0.7-4.2)	.23	1.07 (0.31-3.72)	.91
**Age group (years)**				
60‐69	Reference		Reference	
≥70	1.4 (0.56-3.4)	.48	1.4 (0.5-4.09)	.47
**Education, n (%)**				
No formal education	Reference		Reference	
Up to 10th standard	0.47 (0.18-1.19)	.11	0.49 (0.16-1.45)	.20
12th and above	0.5 (0.06-5.36)	.61	0.57 (0.04-7.40)	.67
**Occupation, n (%)**				
Retired	Reference		Reference	
Unemployed/home	1.9 (0.56-6.7)	.28	1.98 (0.38-10.14)	.41
Other	0.9 (0.21-4.2)	.95	0.98 (0.17-5.6)	.98
**Marital status, n (%)**				
Married	Reference		Reference	
Unmarried, divorced, separated, widow, widower	1.02 (0.39-2.67)	.96	0.68 (0.23-1.97)	.48
**Monthly household income^b^, n (%)**				
<30,000	Reference		Reference	
>30,000	0.84 (0.33-2.12)	.71	0.94 (0.32-2.77)	.92
**Health insurance**				
Yes	Reference		Reference	
No	1.3 (0.55-3.4)	.50	0.71 (0.24-2.03)	.52
**Duration of diabetes mellitus (years)**				
0‐10	Reference		Reference	
≥10	1.4 (0.52-3.8)	.51	1.56 (0.52-4.68)	.42
**Hypertension (years)**				
Yes	Reference		Reference	
No	1.9 (0.76-4.8)	.10	2.09 (0.79-5.48)	.13
**Previous visit to the eye facility**				
Once	Reference		Reference	
Never	0.63 (0.19-2.09)	.46	0.54 (0.14-2.04)	.37

a OR: odds ratio.

b INR: Indian rupee. 30,000 INR=US $350.

### Telephone Follow-Up: Assessing Referral Adherence Rates and Reasons

Participants with ungradable images or diagnosed with referable DR (n=136) were contacted by phone 1 month after screening. The most commonly reported barriers were categorized as other factors (n=70, 51.5%), logistical challenges (n=31, 22.8%), and attitudinal issues (n=27, 19.9%).

Regarding attitude, 17 (12.5%) participants believed their eyes were fine, while logistical obstacles included lack of family support (n=21, 15.4%) and family problems (n=8, 5.8%). Financial constraints, particularly high treatment costs, affected 4 (2.9%) participants. Additionally, 4 (2.9%) were unaware of the importance of DR treatment. Seasonal harvesting (n=14, 10.3%) and other health issues (n=9, 6.6%) were also contributing factors (Table 3).

**Table 3.** Reasons for nonadherence to referral recommendations.

Categories and barriers to referral adherence		Participants (n=136), n (%)
**Attitude**		
	Eyes are fine	17 (12.5)
	Not willing to treatment	3 (2.2)
	Lack of concern about diabetic retinopathy	3 (2.2)
	Not interested in treatment	4 (2.9)
**Financial**		
	High treatment cost	4 (2.9)
**Awareness**		
	Not aware that DR treatment is important	4 (2.9)
**Logistical support**		
	Lack of family support	21 (15.4)
	Travel distance to a health facility	2 (1.6)
	Family problems	8 (5.8)
**Other**		
	Harvesting seasons	14 (10.3)
	Work commitments	4 (2.9)
	Out of station	6 (4.4)
	Phone with family members	3 (2.2)
	Planning to visit a health facility soon	2 (2.2)
	Other health issues	9 (6.6)
	Weather conditions	1 (0.7)
	Technical error (wrong contact numbers, switch-offs, unavailability)	31 (22.8)

### Qualitative

#### Characteristics of the Participants

In total, 28 in-depth interviews were conducted with 9 older adult people with DM and 19 HCPs. Of the 9 people with DM, 4 (44%) were male and 5 (56%) were female. Most (n=7, 78%) were aged 60‐69 years, and 8 (89%) were married. Two-thirds (n=6) lacked formal education, and 6 (67%) were unemployed or engaged in home duties (Tables S6 and S7 in Multimedia Appendix 1). The HCP group included 1 retina specialist (5%), 4 ophthalmologists (21%), 2 optometrists (10%), 3 CHOs (16%), and 9 ASHA workers (47%). Among 19 health care providers, 13 (68%) were female and 6 (32%) were male. Most (n=12, 63%) were aged 31‐40 years.

#### Barriers Associated With Low Referral Adherence

##### Overview

**Figure 2.**
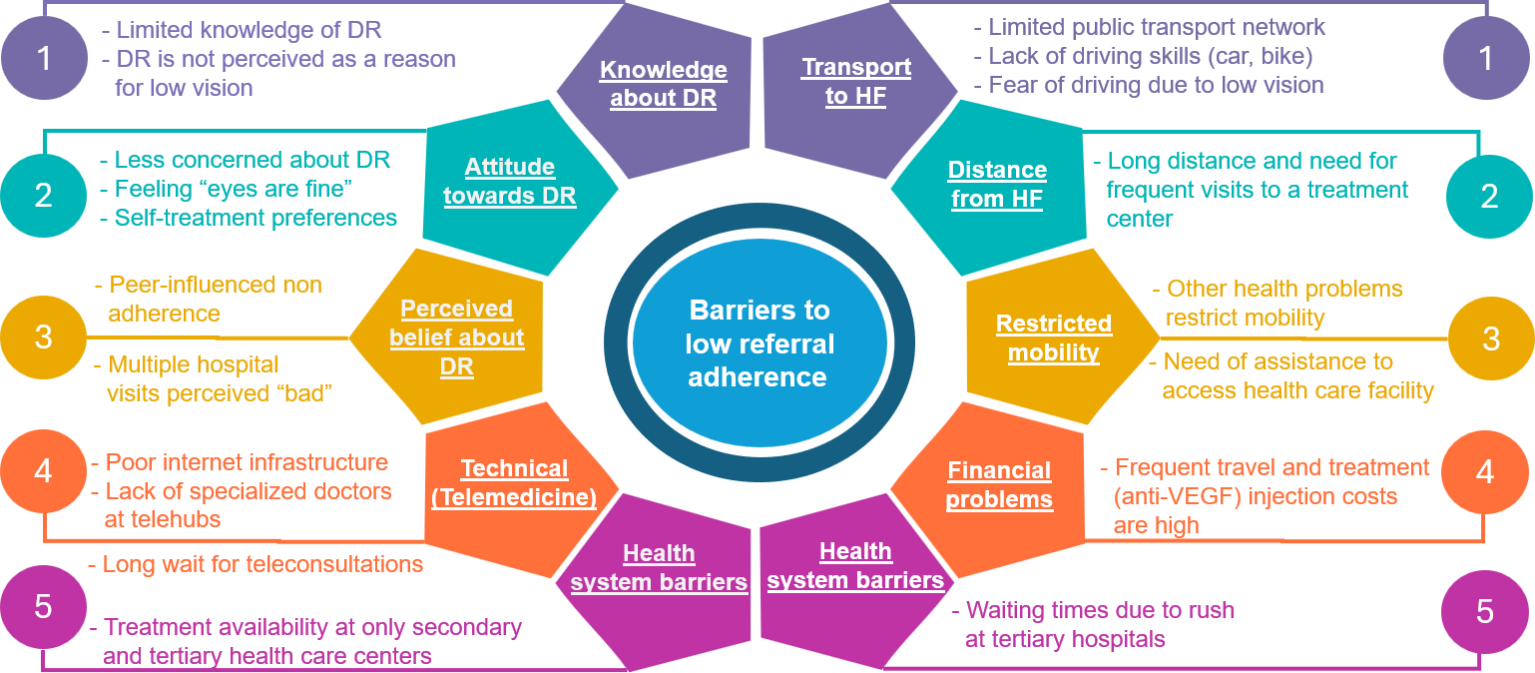
Barriers to referral nonadherence. DR: diabetic retinopathy; HF: health facility; VEGF: vascular endothelial growth factor.

The data revealed three key themes that best explained the barriers to referral adherence: (1) awareness and knowledge-related obstacles, (2) logistical support challenges, and (3) health care system limitations. The results of the in-depth interviews with the stakeholders complemented the quantitative findings of nonadherent participants and shed further light on their findings (Figure 2). The thematic analysis suggested a range of factors that impact adherence to referral instructions and put low adherence or nonadherence into perspective. The quotes under the categories are available in Table S8 in Multimedia Appendix 1.

##### Awareness and Knowledge-Related Barriers

The HCPs observed that patients with DR often delayed treatment due to a lack of awareness about the condition and its complications, frequently mistaking vision changes for aging, cataracts, or simply needing new glasses. These misconceptions often lead patients to request remedies such as eye drops or new glasses, mistakenly believing these will resolve their vision problems. One patient recounted an interaction with fellow patients who had undergone extensive treatments and incurred substantial expenses without experiencing any improvement in vision. This perception highlights a common gap in patient knowledge. Individuals often rely excessively on peer anecdotal evidence, hindering adherence to evidence-based medical treatments. This issue is further complicated by a limited awareness of the crucial role regular follow-up appointments play in managing DR, resulting in poor compliance with treatment protocols. Additionally, some patients tend to avoid health care facilities under the false assumption that their ocular condition has stabilized while engaging in self-treatment, which introduces significant risks and compromises effective disease management.

##### Logistical Support Barriers

###### Mobility and Transportation

The HCPs said that the lack of public transport restricts participants from accessing local transport options for treatment, making it hard for patients to reach the facility for their appointments. One patient with a 2-wheeler reported difficulty driving alone because of their low vision. Longer distances to treatment facilities also decreased the frequency of visits, resulting in higher dropout rates despite initial engagement in treatment. Sometimes, multiple health issues can limit mobility and hinder access to eye care services, leading individuals to prioritize managing other health conditions over maintaining regular DR treatment appointments. This preference reflects a practical decision based on immediate health needs and logistical challenges posed by mobility restrictions rather than a lack of recognition of the importance of DR care.

###### Financial

Cost presents a formidable barrier, restricting many from affording regular travel to treatment centers. Initially, patients may adhere to treatment plans diligently, but as time progresses, dropout rates escalate. A patient articulated their predicament: “I was referred to another hospital for injections, which are financially out of reach due to the distance from home.” The ophthalmologist and retina specialist underscored the substantial financial burden of DR treatment, specifically anti-VEGF (vascular endothelial growth factor) injections, which significantly impacts adherence. Financially disadvantaged patients frequently prioritize other expenses over necessary visits to eye care facilities, thereby exacerbating the challenge of managing DR effectively and consistently. This underscores the critical need for accessible and affordable eye care solutions to mitigate the economic barriers hindering patient care and treatment adherence.

### Health Care System Barriers

#### Burdened Health System

The tertiary eye care hospital experiences high demand, resulting in delayed appointments that led many patients to discontinue treatment after 1-2 visits. Long queues also discourage patients from seeking care at larger eye care centers, underscoring the systemic challenges that affect access to DR treatment services. The large number of patients and extended waiting times pose significant challenges for individuals trying to attend appointments and receive timely treatment.

#### Technical Challenges

Telemedicine facilities are beneficial for extending eye care access [49], but they face implementation challenges. The limited availability of specialized doctors and unreliable internet in peripheral areas hinder teleconsultations. Village residents encountered prolonged wait times at telemedicine centres and expressed reluctance to wait for consultations with doctors at these facilities.

## Discussion

### Principal Findings

This study investigated barriers to referral adherence after AI-enabled DRS at primary (community) and secondary (CHC) sites among those who did not follow referrals to district hospitals for further management. We implemented AI-enabled DRS programs for older adults in public health settings in Punjab, India, during the COVID-19 pandemic. AI has been promoted as a transformative tool in health care to help attain health and health-related Sustainable Development Goals among older adults [50,51]. However, the impact of implementing AI on patient outcomes depends on many factors beyond diagnostic accuracy, including care access, referral adherence, and the implementation of treatment and management recommendations [52]. In our study, only 23 of 159 (14.5%) patients screened adhered to referral recommendations. Of these, only 9 visited the district hospital, while the others chose different facilities, indicating various factors influencing their decision-making regarding referral locations [53]. This finding contrasts with a study conducted in South India examining the factors influencing the utilization of referral services from secondary to tertiary eye care, which reported noncompliance rates of 31.65% and 24.2%, respectively [33].

Telephone interviews identified key barriers in our study, including low awareness about DR (n=4, 2.9%), attitudinal issues (n=27, 19.9%), and a lack of family support (n=21, 15.4%). Economic 27 (16.4%) and attitudinal barriers (44.2%) to referral service access were consistent with findings by Padhy et al [33] Our analysis showed no differences in the characteristics of adherent and nonadherent participants. However, nonstatistically significant differences between adherent and nonadherent groups do not imply that these factors are irrelevant or that there are no relationships. This indicates that we did not detect significant associations with the current sample size and variability. However, the wide confidence intervals suggest additional quantitative data are needed to improve estimate precision.

Nevertheless, we have no evidence that demographic and health variables alone are sufficient to understand or predict adherence behavior in patients with DR. Technical errors (Table 3) prevented us from contacting 31 of 136 (22.8%) participants, potentially impacting our quantitative analysis, including the univariate and multivariate analysis results. The unprecedented COVID-19 pandemic may have also negatively affected health-seeking behavior and treatment adherence [19,22].

We prioritized incorporating insights from key stakeholders through qualitative, in-depth interviews to guide informational power. Our study found that older adult individuals lack adequate awareness about DR complications, resulting in misconceptions about the causes of vision changes. Lack of awareness discourages service use, enabling unchecked harmful conditions and potentially leading to adverse outcomes due to delayed care-seeking. Further, the participants encountered difficulties affording transportation and treatment expenses and taking time off work for medical appointments, driven by concerns about their daily income [54]. Additionally, attitudes such as believing their eyes are healthy and logistical challenges like lack of family support impeded referral adherence [23]. Reliance on anecdotal evidence (do not go for eye treatment) from peers discouraged adherence to referral recommendations. Local and regional factors, such as the peak harvesting season (Table 3), hindered older adult adherence to referral recommendations (n=14, 10.3%). In-depth stakeholder interviews complemented the telephone follow-up findings, revealing barriers to referral recommendations. Similar programs, like the point-of-care DR examination program initiative, reported suboptimal follow-up rates despite targeted interventions, highlighting the need for more personalized strategies to enhance adherence [24]. Community-based programs have proven effective in enhancing access to eye care and improving patient adherence to continuity of care recommendations [24,55]. Bonilla-Escobar et al [24] showed higher adherence to referral recommendations when personalized approaches, such as phone calls, voicemails, and result letters with appointment details, were used. Transportation interventions, including bus passes, taxi vouchers, reimbursed travel, and tailored services, enhance health outcomes in chronic diseases, particularly for older adults and women [56].

Teleophthalmology, for instance, has accelerated health care access in rural and remote areas [57], overcoming significant travel constraints, time constraints, and economic barriers through community-based program initiatives [58,59]. However, the HCPs noted that technical challenges, such as underdeveloped internet infrastructure in rural areas, pose challenges to implementing teleophthalmology programs [60]. Hence, it is critical to identify methods for establishing DRS programs tailored to available resources and health care infrastructure [24,61]. In our study, older adult participants highlighted financial constraints as barriers to accessing referral services, a sentiment corroborated by the HCPs’ in-depth interviews. The findings align with existing literature on economic challenges among individuals with visual impairment in developing countries [62]. The high costs of DR treatment often compel financially disadvantaged patients to prioritize other essential expenses over necessary eye care. Patients and HCPs should be informed about the comprehensive coverage offered by the Ayushman Bharat Pradhan Mantri Jan Arogya Yojana public health insurance program in India, including DRS and treatment. This ensures adequate access to necessary services through insurance benefits [63].

Our study findings revealed issues in the care process in rural areas, such as patients self-referring and bypassing the referral system hierarchy [64]. Systemic challenges such as delayed appointments and long queues discourage patients from continuing treatment beyond 1 or 2 visits due to access issues [23]. The World Report on Vision has recommended an integrated care model incorporating primary, secondary, and tertiary care, ensuring clear roles and appropriate referrals at each health care system level [65].

This study’s strength lies in triangulating quantitative findings with qualitative insights to address research questions regarding referral adherence. Additionally, conducting the study across primary (community) and secondary (CHC) health care systems provides comprehensive information on referral adherence within a network of public health systems.

A limitation of this study is its focus on a public health network in North India within one state, potentially limiting the generalizability of its findings to broader settings. Potential biases from self-reported data during telephone follow-ups may lead to underreporting or overreporting adherence based on perceived expectations and limited generalizability due to the study’s regional focus. The study was conducted during the COVID-19 pandemic, marked by significant disruptions to health care systems and changes in patient behavior. These unique circumstances, including the impact of COVID-19 on decision-making regarding nonemergency medical care, may have introduced factors related to health care access that are specific to this time. Furthermore, nearly 22.8% of screened patients (Table 3) were not successfully contacted despite multiple attempts, which would increase the imprecision of estimates yielded by the regression analysis. For example, only 23 adherent participants contributed data to the multivariate model and therefore the analyses may be hindered due to issues related to statistical power and imbalanced group sizes [66]. This study did not extensively collect detailed information on health care system factors affecting adherence, such as hospital waiting times, hospital accessibility, and staff attitudes [33]. This highlights the necessity of exploring additional factors that influence adherence, including psychological, social, and systemic barriers [23,67].

In this pilot study, approximately 390 of 948 (41%) participants were recruited due to COVID-19 pandemic restrictions. Additionally, the limited sample size in the nonadherent group and high variability in the collected data resulted in insufficient statistical power to accurately identify determinants of nonadherence, leading to large confidence intervals and reduced precision in the estimates.

### Conclusion and Policy Implications

This study, conducted during the COVID-19 pandemic, has demonstrated suboptimal adherence among older adult patients to referral recommendations. Participants considered barriers such as knowledge gaps, logistical challenges, and health system–related issues to have hindered referral adherence. Given the unique context of the pandemic, the results should be generalized with caution. Future studies should consider pragmatic trial study designs incorporating implementation science approaches using mixed methods to examine barriers to referral and adherence across diverse contexts. This could include exploring community beliefs and other social determinants influencing service uptake. Policy-level changes are essential to integrating teleophthalmology into public health programs and improving infrastructure for AI-enabled DRS, enhancing access and outcomes. An effective, well-coordinated referral system with defined protocols, streamlined communication, reliable transportation, trained personnel, integrated systems, and collaboration may address the challenges in the referral systems [68,69].

## Supplementary material

10.2196/67047 Multimedia Appendix 1 Supplementary materials.

## Abbreviations

ASHA: Accredited Social and Health Activists
CHC: community health center
CHO: community health officer
DM: diabetes mellitus
DR: diabetic retinopathy
DRS: DR screening
HCP: health care provider
VTDR: vision-threatening diabetic retinopathy
