# Supplementary material for: Barriers and Determinants of Referral Adherence in AI-Enabled Diabetic Retinopathy Screening for Older Adults in Northern India During the COVID-19 Pandemic: Mixed Methods Pilot Study
Source: JMIR Form Res. 2025 Mar 31;9:e67047. doi: 10.2196/67047 (PMC11975122; doi:10.2196/67047)
Supplement: Multimedia Appendix 1 [file formative-v9-e67047-s001.pdf]

Barriers and Determinants of Referral Adherence in AI-Enabled Diabetic Retinopathy Screening for the Elderly: Insights from a Pilot Study in Northern India

Selection criteria for the healthcare providers according to delivery of services

Supplementary Table 1 outlines the delivery of services in the NPCBVI program. It has been adapted from the NPCBVI and Operational Guidelines for Primary Eye Care at the Health and Wellness Centre (A Part of Comprehensive Primary Health Care).

Supplementary table 1: Selection criteria for the healthcare providers

**Health Facility**	**Packages of services**	**Responsibilities**
Community	Screening, preventive care activities, promotion of eye care, and home-based follow-up<br>Screening for blindness and Refractive errors<br>Community screening for congenital disorders referral	ASHA
HWC	Screening for blindness and refractive error<br>Screening for visual acuity in Diabetic patients<br>Create awareness through IEC to avoid and prevent eye diseases like (trachoma, xeropthalmia, injury, etc.)<br>Referral for further review and treatment	CHO/ANM/MPW
PHC	diagnosis and regular treatment for common eye conditions<br>Refer for advanced treatment (low vision, glaucoma, cataract)<br>**For DR**<br>Screening for DR and facilitating consultation with an eye specialist at an early stage, an annual eye examination is a must for a diabetic	Ophthalmic officer (Optometrist)

	patient, fundus imaging if a camera is available, and Referral for further treatment.	
DH	Screening and treatment<br>Refer for further treatment	Ophthalmologist<br>Ophthalmic officer
Tertiary care centre	Advanced DR diagnosis and treatment	Retina specialist<br>Ophthalmic officer (Optometrist)

ASHA - Accredited Social Health Activist, ANM - Auxiliary Nurse Midwife, CHC - Community Health Centre, DH - District Hospital. HWC- Health and Wellness Centre, DR - Diabetic retinopathy, IEC - Information Education and Communication, PHC - Primary Health Centre

Supplementary table 2: Inclusion criteria for study participants

Inclusion criteria for PwDM	Inclusion criteria for HCP
Male or female, aged 60 years or older	Male or female, with an experience of 3 years or more in the healthcare system
Diagnosed DM irrespective of duration of diabetes	Involved in DM and DR screening, diagnosis, and treatment in public health systems (community, primary health centre, and tertiary healthcare centre) (supplementary table 4)
Able to communicate in Hindi or Punjabi	Able to communicate in Hindi, Punjabi or English

***DM: Diabetes Mellitus, DR - Diabetic Retinopathy, HCP - Healthcare Provider, PwDM - People with Diabetes Mellitus

Supplementary 3: Comprehensive list of guideline questions

Questions for elderly participants:

- Can you describe your understanding of diabetic retinopathy and its potential complications? (Probe: DR as a reason for low vision, blindness)
- Were you informed about the importance of following up on your screening referral? What was the information provided? (Probe: importance of referral, treatment)
- What challenges did you face for not visiting the healthcare facility as advised during your DRS procedures? What discouraged you from adhering to the referral advice? (Probe: personal, financial, social, past experiences with the health system)
- Were there any concerns about the treatment or the facility that affected your decision to follow through?

Questions for healthcare providers:

- Have you encountered specific barriers elderly patients report when adhering to referrals? (probe: awareness,
- How do you ensure patients understand the importance of the referral? What challenges do you face in communicating this to elderly patients?
- Are there systemic or logistical challenges in the referral process (Probe: wait times, access to specialists)?
- What improvements could be made to the referral system to improve adherence among elderly patients?
- Do you think there is a gap in addressing patients concerns during the referral process?

Supplementary Table 4: Sociodemographic variables of the study participants

Variable	Health facility-based screening (n=176)	Community-based screening (n=214)	Total (n=390)
Gender, n(%)			
Male	93 (52.8)	83 (38.8)	176 (45)
Female	83 (47.2)	131 (61.2)	214 (55)
Age			
60-69	113 (64.2)	131 (61.2)	244 (62.6)
≥70	63 (35.8)	83 (38.8)	146 (37.4)
Age (mean ± SD)	66.2 ± 5.8	68.1 ± 6.5	67.2 ± 6.2
Education, n (%)			
No formal education	78 (44.3)	111 (52)	189 (48.5)
Upto 10th standard	89 (50.5)	90 (42)	179 (45.9)
12th & above	9 (5.2)	13 (6)	22 (5.6)
Occupation, n (%)			
Retired from service	14 (8)	40 (18.7)	54 (13.8)
Unemployed/Home duties	126 (71.6)	146 (68.2)	272 (69.7)
Others	36 (20.4)	28 (13)	64 (16.5)
Marital status, n (%)			
Married	150 (85.2)	139 (65)	289 (74)
Un/Div/Sep/wid/er	26 (14.8)	75 (35)	101 (26)
Household monthly income, n (%)			
<30000	147 (85.5)	122 (57)	269 (69)
>30000	29 (16.5)	92 (33)	121 (31)
Health insurance			
Yes	47 (26.7)	75 (35)	122 (31.3)
No	129 (73.3)	139 (65)	268 (68.7)

Duration of diabetes (years), n (%)<br>0 - 10<br>>10	 <br>133 (75.6)<br>43 (24.4)	 <br>146 (68.2)<br>68 (31.8)	 <br>279 (71.5)<br>111 (28.5)
Duration of diabetes (mean ± SD)	7.3 ± 6.7	8.6 ± 7.18	8.03 ± 6.9
Hypertension (years)<br>Yes<br>No	 83 (47)<br>93 (53)	 121 (56.5)<br>93 (43.5)	 204 (52.3)<br>186 (47.7)

DM: Diabetes Mellitus, INR: Indian National Rupee, Un/Div/Sep/widow/er: Unmarried, divorced, widow, widower

Supplementary Table 5: Treatment status of the adherent group participants

**Treatment status of adherent elderly group**	**n = 23**
Visited a charitable hospital, but no treatment was available	1
Visited a charitable hospital, but no treatment was available	1
Visited a district hospital, and eye drops were given	1
Visited a private hospital in Moga, but no treatment was given	1
Visited a private hospital in Moga, but no treatment was given	1
Visited a private hospital in Hoshiarpur, but no treatment was given	1
Visited a district hospital, advised cataract surgery	1
Visited a district hospital, advised diabetic medicine	1
Visited a district hospital, advised glasses	2
Visited a private hospital, but no treatment was given	1
Visited a district hospital, given next appointment	2
Visited a district hospital, but no treatment was given	1
Visited a private hospital, but no treatment was given	1
Visited a district hospital, given eye drops	1
Not sure about the hospital name	1
Visited a private hospital, cataract surgery done	3
Visited a private hospital, appointment given for cataract surgery	1
Visited a district hospital, cataract surgery done	1
Visited a district hospital, and an appointment given for cataract surgery	1

DH: District Hospital

Note: Total 231/390 (50.2%) were not referred, 159/390(40.8%) participants were referred, 23/159(14.5%) followed the advice and visited a health facility and is presented in the supplementary table above.

Supplementary Table 6: Characteristics of people with diabetes mellitus (PwDM)

**Sociodemographic Characteristics of PwDM**	**n=9**
**Gender, n (%)**	
Male	4 (44.4)
Female	5 (55.6)
**Age, n (%)**	
60-69	7 (77.8)
≥70	2 (22.2)
**Marital status, n (%)**	
Married	8 (88.9)
Un/Div/Sep/wid/er	1 (11.1)
**Education, n (%)**	
No formal education	6 (66.7)
Upto 10th standard	2 (22.2)
12th & above	1 (11.1)
**Occupation, n (%)**	
Retired from service	1 (11.1)
Unemployed/Home duties	6 (66.7)
Others	2 (22.2)
**Duration of Diabetes**	
Mean ± SD (yrs)	11.9 ± 6.3

Supplementary table 6: Characteristics of Healthcare providers (HCP)

Characteristics of HCPs	n=19
**Gender, n (%)**	
Male	6 (31.6)
Female	13 (68.4)
**Age (years), n (%)**	
31-40	12 (63)
41-50	7 (37)
**Professional status, n (%)**	
Retina specialist	1 (6.2)
Ophthalmologist	4 (21)
Optometrist	2 (10.5)
CHO	3 (15.8)
ASHA	9 (47.4)

***ASHA - Accredited Social Health Activist, CHO - Community Health Officer; HCP - Health Care Provider

Supplementary Table 8: Themes and examples of stakeholder's quotations

Patient and Healthcare providers

Theme	Highlights	Quotes
**Awareness and knowledge-related barriers**		
DR-specific awareness and knowledge barriers	• Patients are unaware of DR, its complications, and risk factors<br>• Feeling "eyes are fine"<br>• Self-treatment preferences<br>• DR is not perceived reason for low vision<br>• Peer-influenced non-adherence<br>• Frequent hospital visits seen negatively	*"Due to limited awareness about diabetic retinopathy complications, patients often struggle to adhere to their treatment plans."* CHO3<br><br>*"Patients often delay diabetic retinopathy treatment, mistaking vision changes for aging, cataracts, or needing new glasses rather than recognizing the actual underlying cause."* Optom 1<br><br>*"They say, give us some eye drops or spectacles* ***(wo itna he bolte hai thoda sa number de do jo hota hai"). We are happy with whatever vision we have".*** Optom2<br><br>*"Patients due to lack of knowledge refrain from visiting health facilities, believing their eyes are fine, and resorting to self-treatment, which can be dangerous."* Optom1<br><br>*"The patients do not know that diabetic retinopathy treatment usually requires frequent follow-ups. When we explain this to patients, they often lose interest and stop visiting the health facility."* Oph1
**Logistical support barriers**		
Mobility and transportation	• Limited public transport network<br>• Fear of driving alone<br>• Long distance<br>• Restricted mobility	*"Accessing local transport for treatment is problematic, as limited public transport buses are available in this area, making it difficult to travel to (name of the facility) for appointments." CHO1*<br><br>*"Local transport is an issue. There are few public transport buses, so traveling to (name of the facility) becomes very difficult". P3*<br><br>*"While driving, whenever the light would fall in the eyes, it would disperse* ***("Jab light aankhon me girti thi to failti thi"),*** *and this happens when I used to step out in the sun. I fear driving alone now*". P1

		Many struggles to afford frequent travel to treatment centres. While they may start treatment well, dropout rates increase over time. Opt2<br><br>*"I cannot visit the hospital regularly for my eye treatment due to my multiple health problems consuming most of my time."* P6<br><br>*"I can no longer walk to the hospital due to knee pain, and I need someone to accompany me."* P5
Financial	• Frequent facility visits and cost of treatment	*"Costing is a big factor; people cannot afford to travel frequently via any means to the treatment centres. So, initially, they start well, and then there is a dropout".* Oph1<br><br>*"I was referred to another hospital, where they informed me that I needed injections that will be available at the tertiary hospital located at a distance from home, requiring frequent travel, which I cannot afford."* P 6<br><br>"*Diabetic retinopathy treatment is costly; when a patient requires anti-VEGF injection, poor patients cannot afford that, so they avoid visiting the health facility."* Optom1
**Healthcare system barriers**		
**Burdened health system**	• Waiting time at treatment facilities<br>• Treatment availability only at secondary and tertiary facilities	*"Due to the high rush at the tertiary hospital (name of the hospital), they receive appointments for later dates and often drop out after one or two appointments."* RS1<br><br>*"Patients dislike standing in long queues and avoid visiting larger healthcare centres."* CHO3
**Technical challenges**	• Poor internet infrastructure<br>• Lack of specialized doctors at telehubs	*"There are few specialized doctors at the Tele Hubs for tele consultations, and connecting with them through the patchy internet in peripheral areas is very difficult."* CHO 1<br><br>*"Village residents find it difficult to sit for long periods for teleconsultations. They often mention having more chores, such as gathering fodder for the cattle."* CHO 2

DR: Diabetic retinopathy, CHO: Community Health Officer, Oph: Ophthalmologist, Optom: Optometrist, P: Patient, RS: Retina Specialist, VEGF: Vascular Endothelial Growth Factor
